# Supplementary material for: Impact of Feeding Probiotics on Blood Parameters, Tail Fat Metabolites, and Volatile Flavor Components of Sunit Sheep
Source: Foods. 2022 Aug 31;11(17):2644. doi: 10.3390/foods11172644 (PMC9455658; doi:10.3390/foods11172644)
Supplement: Supplementary file 1 [file foods-11-02644-s001.zip › Supplementary Figure S1.pdf]

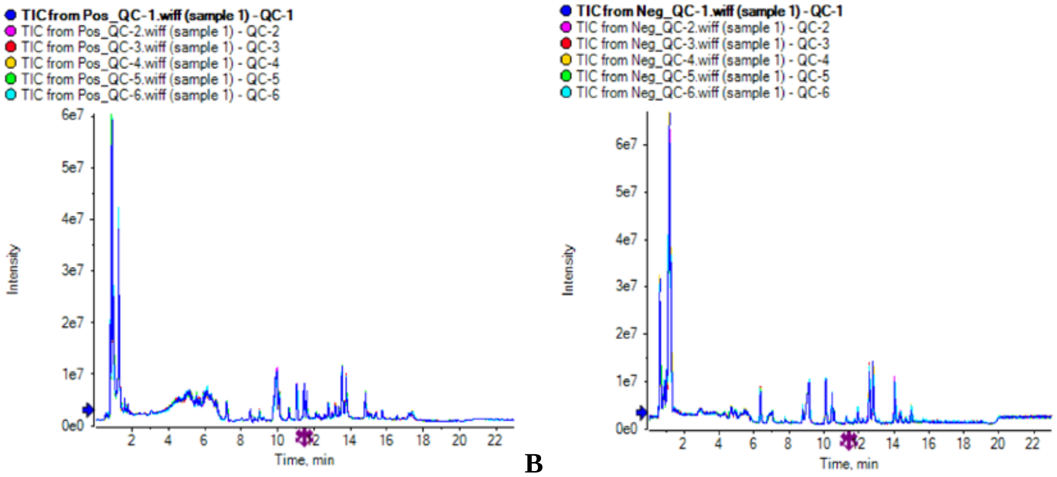

A **B**

Figure S1. Ion mode QC sample total ion chromatogram overlay. The abscissa in the figure represents the retention time of each chromatographic peak, and the ordinate represents the intensity value of the peak. A is the overlay of the total ion chromatogram of the QC sample in positive ion mode, and B is the overlay of the total ion chromatogram of the QC sample in negative ion mode.
