# Supplementary material for: Impact of Feeding Probiotics on Blood Parameters, Tail Fat Metabolites, and Volatile Flavor Components of Sunit Sheep
Source: Foods. 2022 Aug 31;11(17):2644. doi: 10.3390/foods11172644 (PMC9455658; doi:10.3390/foods11172644)
Supplement: Supplementary file 1 [file foods-11-02644-s001.zip › Supplementary Figure S2.pdf]

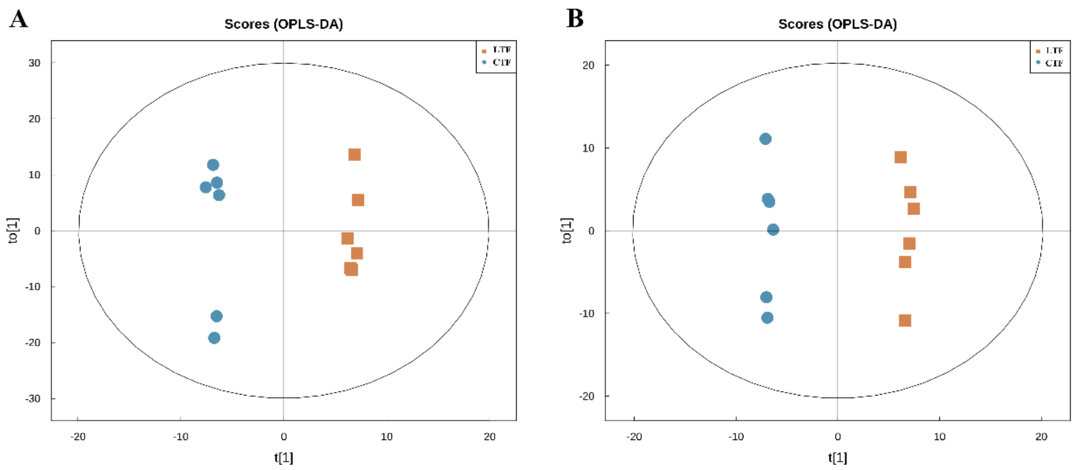


Figure S2. The difference in the OPLS-DA score of tail fat between the two groups. In the figure, t[1] represents principal component 1, to[1] represents principal component 2, and the ellipse represents the 95% confidence interval. The dots of the same color represent each biological replicate within a group, and the distribution of the dots reflects the degree of difference between and within groups. S2A is the positive ion mode OPLS-DA score map, S2B is the negative ion mode OPLS-DA score map.
