# Supplementary material for: Impact of Feeding Probiotics on Blood Parameters, Tail Fat Metabolites, and Volatile Flavor Components of Sunit Sheep
Source: Foods. 2022 Aug 31;11(17):2644. doi: 10.3390/foods11172644 (PMC9455658; doi:10.3390/foods11172644)
Supplement: Supplementary file 1 [file foods-11-02644-s001.zip › Supplementary Figures S3-S5.pdf]

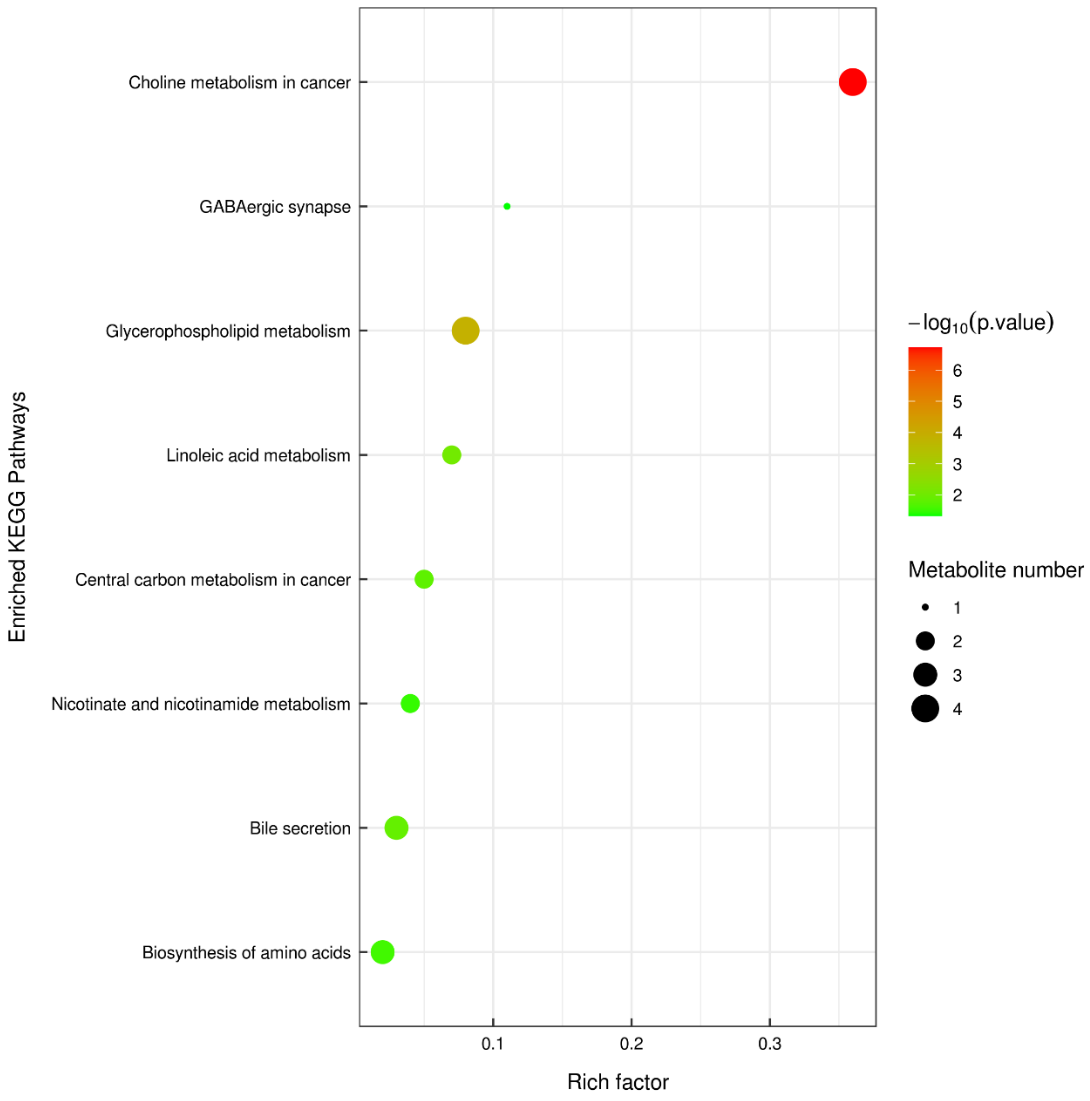

Figure S3. Metabolic pathways with different color dots representing the differential compounds.

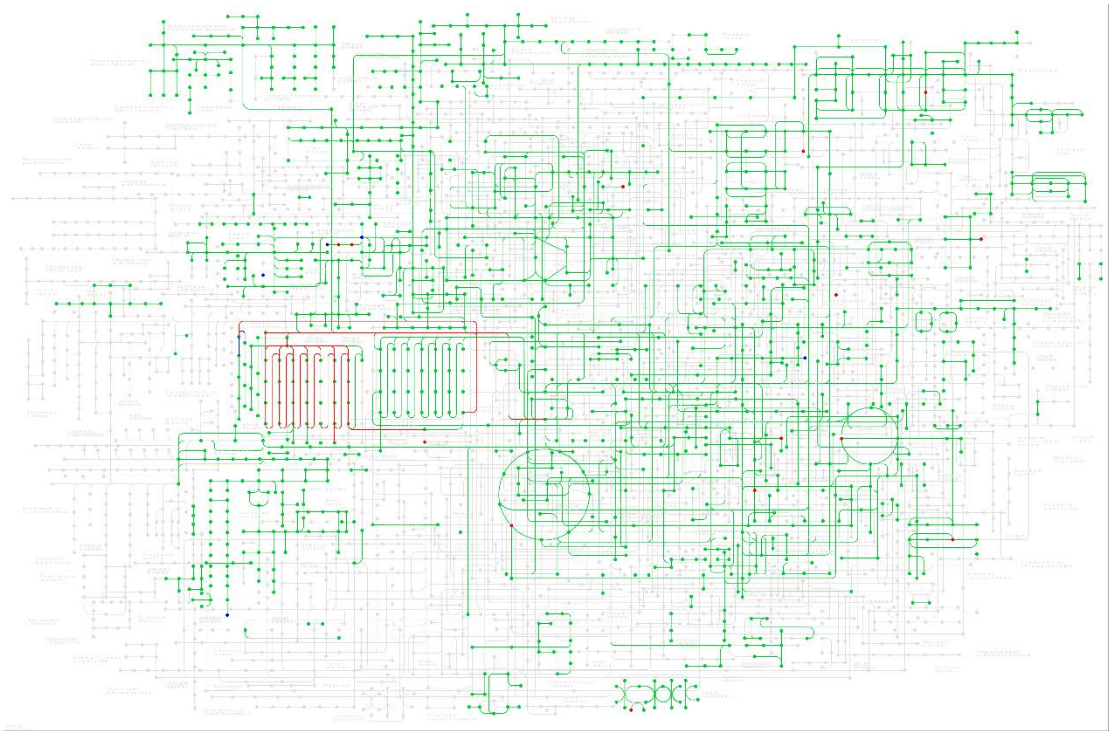

Figure S4. “oas01100”. Metabolic pathways with different color dots representing the differential compounds for the two comparison groups.

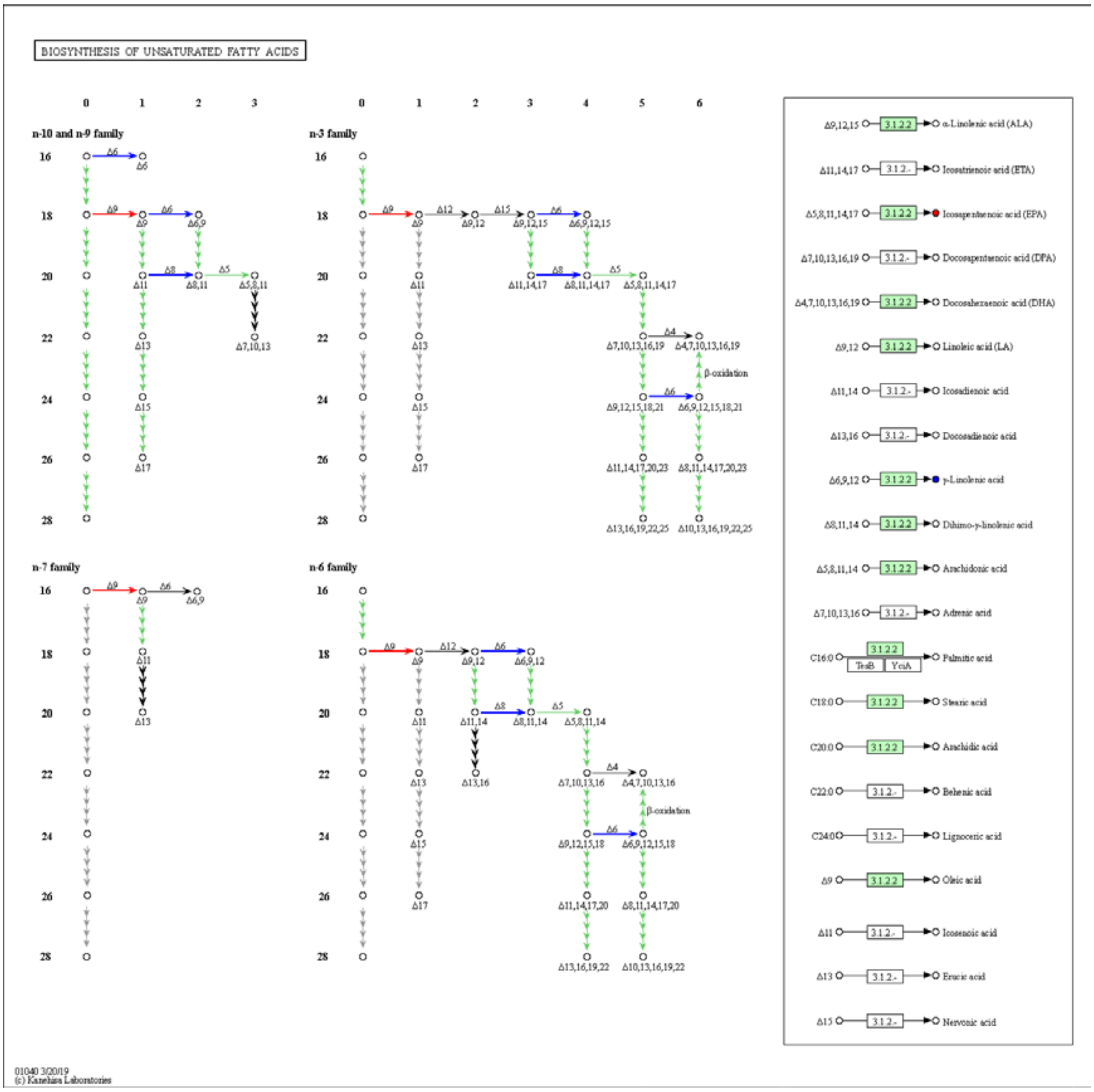

Figure S5. “oas01040”, biosynthesis of unsaturated fatty acids. Red means up-regulation Blue means down-regulation
